# Supplementary material for: Faecal microbiota of schoolchildren is associated with nutritional status and markers of inflammation: a double-blinded cluster-randomized controlled trial using multi-micronutrient fortified rice
Source: Nat Commun. 2024 Jun 18;15:5204. doi: 10.1038/s41467-024-49093-4 (PMC11189458; doi:10.1038/s41467-024-49093-4)
Supplement: Supplementary file 1 — Supplementary information [file 41467_2024_49093_MOESM1_ESM.pdf]

Supplementary information

Supplementary figures 1, 2, 3
Supplementary tables 1, 2, 3, 4, 5, 6, 7

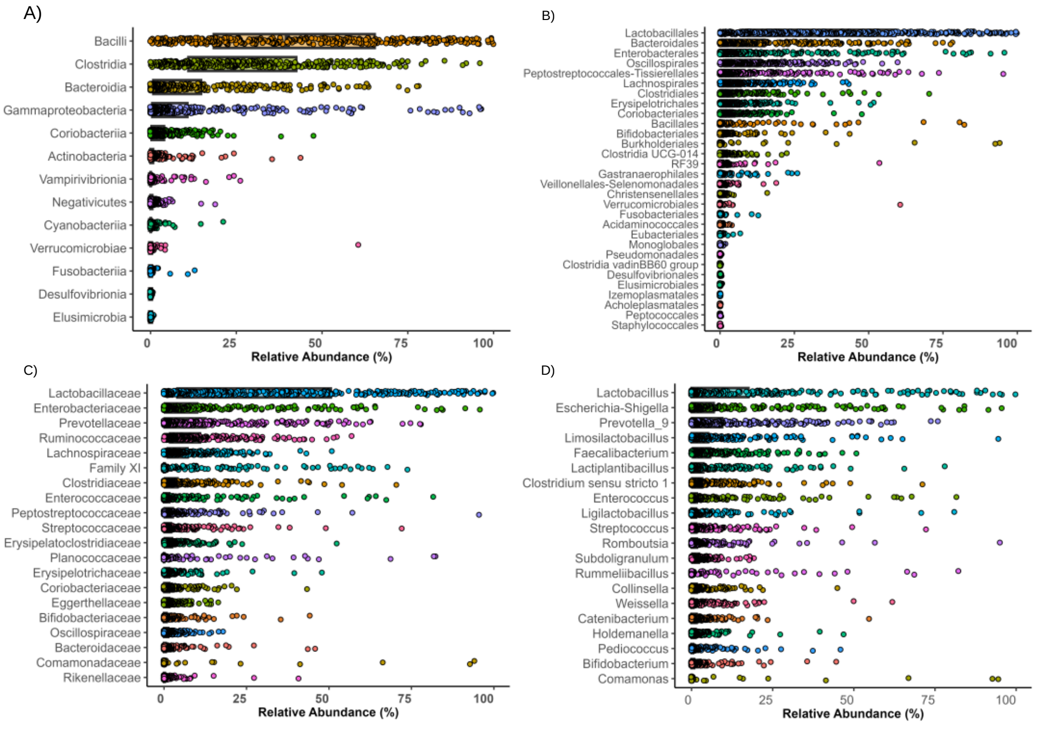

Supplementary Figure 1: Relative abundance of faecal bacteria based on 16S rRNA sequencing data of 380 Cambodian school children at class level (A), order level (B), family level top 20 (C), genus level top 20 (D) Box plot represents median and minimum and maximum. Source data are provided as a Source Data file.

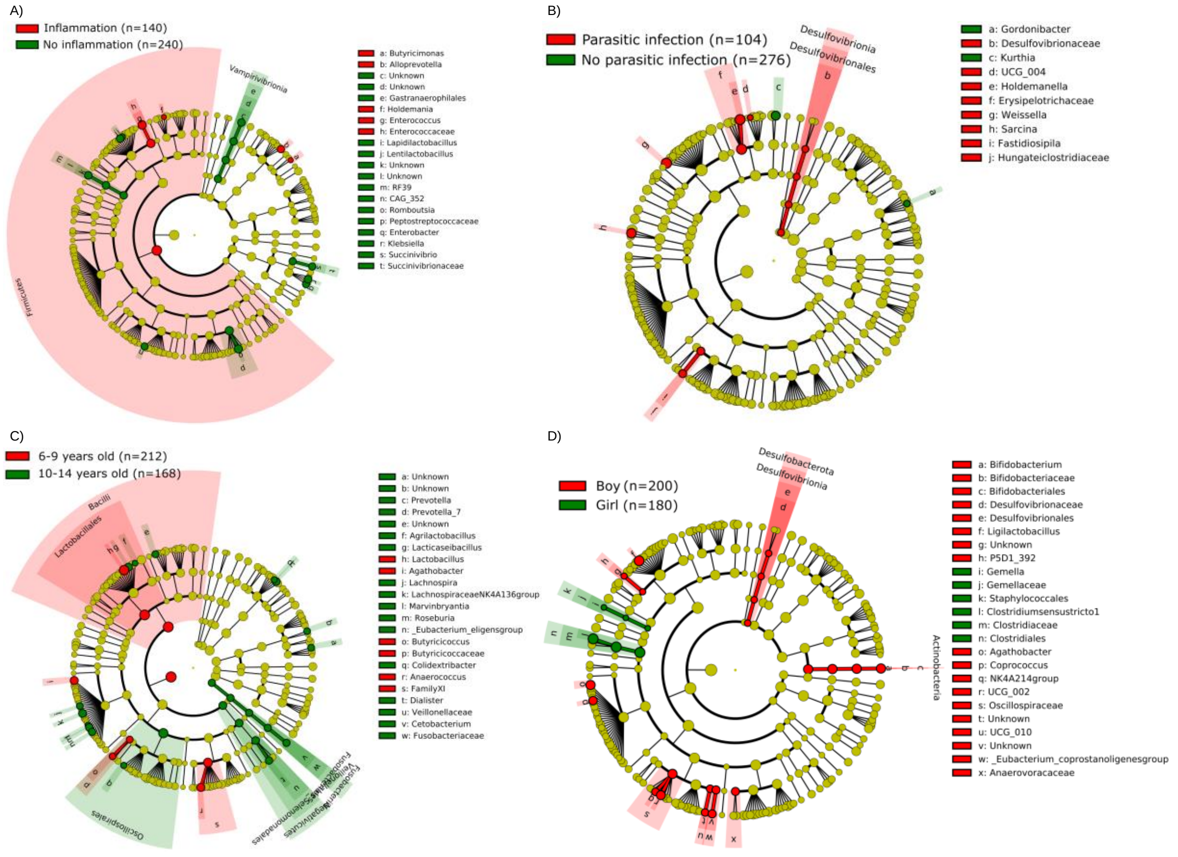

Supplementary Figure 2: Differential ASVs between the children grouped according to A) their systemic inflammation, B) parasite infection, C) age, D) sex. The differences were identified using Linear discriminant analysis Effect Size (LEfSe) analysis with a unadjusted P-value cut-off of < 0.05 and a logarithmic LDA score of > 2. The cladogram representation illustrating taxonomic levels from the innermost phylum ring to the outermost genera ring. Each circle represents a bacterial member within that level. Circles coloured green or red shows significant enrichment of those taxa in the groups indicated by the legend of each individual cladogram. The number of samples in each subcategory is indicated on each graph. Source data are provided as a Source Data file.

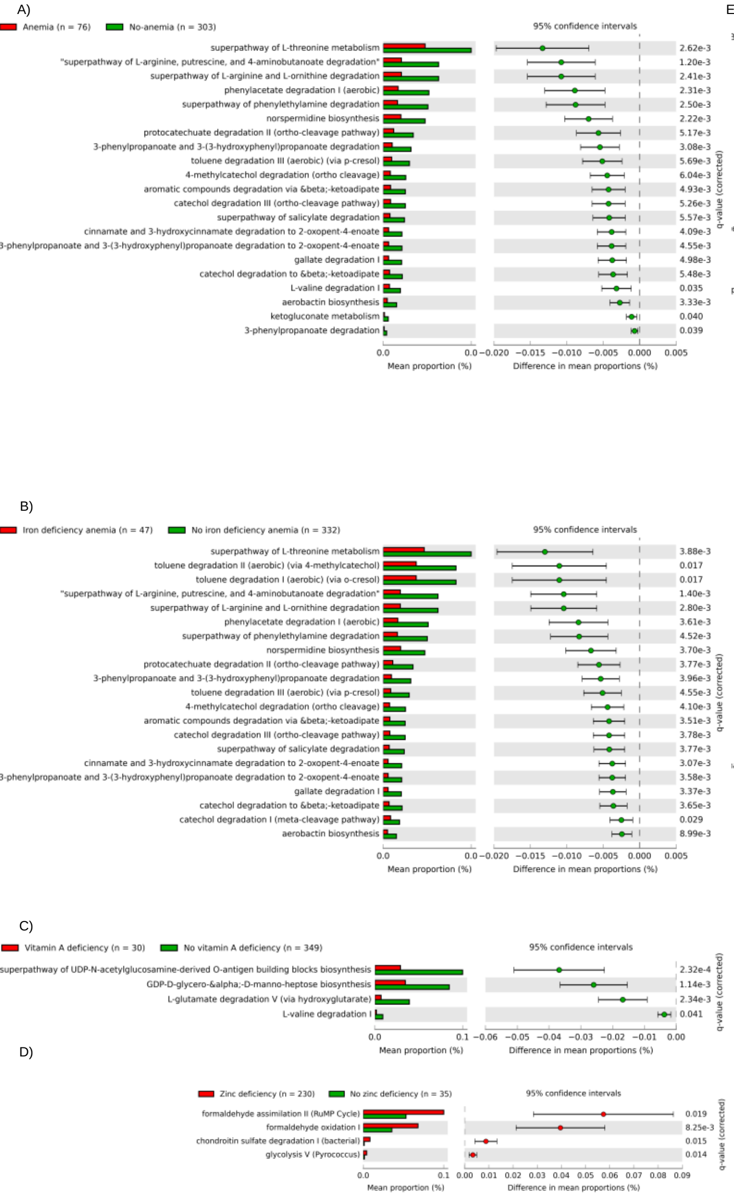

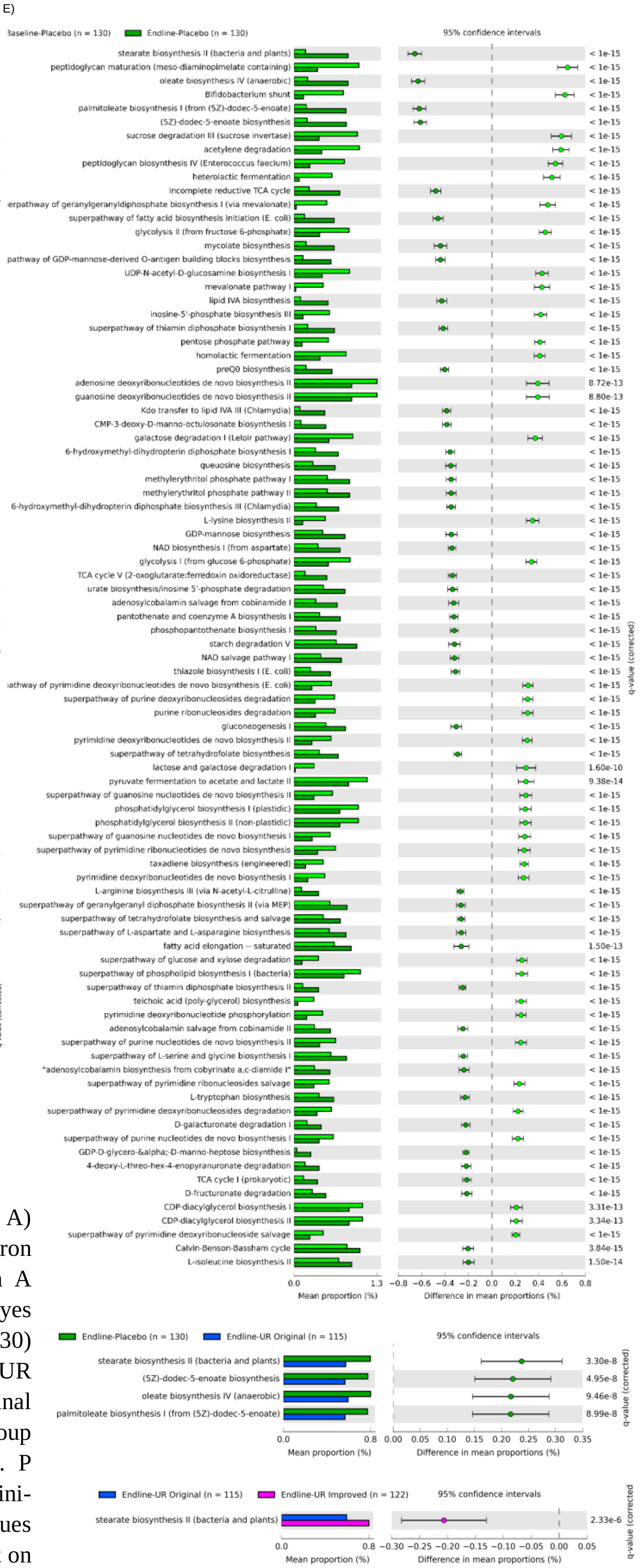

Supplementary Figure 3. Differential KEGG pathways in A) Anaemia (anaemic (n=76) and non-anaemic (n=303)), B) Iron deficiency anaemia (yes (n=47) and no (n=332)), C) Vitamin A deficiency (yes (n=30) and no (n=349)), D) Zinc deficiency (yes (n=230) and no (n=35), E) Baseline and endline Placebo (n=130) with effect size of > 0.2, F) Endline Placebo (n=130) and UR original (n=115) with effect size of > 0.2, G) Endline UR original (n=115) and UR improved (n=122) with effect size of > 0.2. Group significance was measured using a two-tailed Welch's t-test. P values were adjusted for multiple comparison using the Benjamini-Hochberg (BH) False Discovery Rate correction. Adjusted p-values below 0.05 were considered statistically significant. The bar plot on the left shows the mean proportion of each group. The dot plot on the right shows differences in the mean proportion Source data are provided as a Source Data file.

Supplementary Table 1. Baseline characteristics of the participants

	Proportion (%)
**Sex**	n = 380
Boy	52.6
Girl	47.4
**Age group**	n = 380
6 - 9 years	55.8
10 - 14 years	44.2
**Underweight (Yes/No)**	n = 215
Yes	46.5
**Stunting (Yes/No)**	n = 380
Yes	45.3
**Anaemia (Yes/No)**	n = 380
Yes	20
**Iron deficiency (Yes/No)**	n = 380
Yes	50.8
**Iron deficiency anaemia (Yes/No)**	n = 380
Yes	12.4
**Hemoglobinopathy (Yes/No)**	n = 380
Yes	55.5
**Vitamin A deficiency (Yes/No)**	n = 380
Yes	7.9
**Zinc deficiency (Yes/No)**	n = 326
Yes	89.3
**Systemic Inflammation (Yes/No)**	n = 380
Yes	36.8
**Parasite infection (Yes/No)**	n = 380
Yes	27.4
**Gastrointestinal inflammation (Yes/No)**	n = 347
Yes	2.9

Supplementary Table 2: Micronutrient contents of normal (Placebo) rice and the two types of fortified rice (per 100 g dry rice)

Micronutrients	Placebo	UR-original	UR-improved
Iron (mg)*	0.3	10.67	7.55
Zinc (mg)	1.0	3.04	2.02
Vitamin B1 (mg)	ND	1.06	1.43
Folic acid (mg)	ND	0.17	0.28
Vitamin A (mg)	0.03	-	0.64
Vitamin B3 (mg)	ND	-	12.57
Vitamin B12 (µg)	0.4	-	3.8
Vitamin B6 (mg)	0.1	-	-

*: Ferrous glycinate

ND: not determined

- : Not detected

Supplementary Table 3: Impact of the intervention on child growth and micronutrient status

	n	%	Contrasts						Global Effect
			UR improved vs. Placebo			UR Original vs. Placebo			
			OR	CI	*p*	OR	CI	*p*	*p*
Stunting	380	45.3%	0.58	[0.12 - 2.76]	*0.4955*	0.61	[0.12 - 3.04]	*0.5443*	*0.75247*
Anaemia	380	20.0%	0.02	[0.00 - 0.86]	***0.0411***	0.21	[0.01 - 6.94]	*0.3782*	*0.0746*
Iron deficiency	380	50.8%	1.40	[0.55 - 3.52]	*0.4778*	1.94	[0.76 - 4.93]	*0.1652*	*0.3816*
Iron deficiency anaemia	380	12.4%	0.37	[0.04 - 3.47]	*0.3813*	2.44	[0.16 - 36.4]	*0.5177*	*0.3234*
Vitamin A deficiency	380	7.9%	0.10	[0.01 - 0.72]	***0.0223***	8.20	[0.46 - 148.00]	*0.1536*	***0.0045***
Zinc deficiency	326	89.3%	0.03	[0.00 - 0.12]	***< 0.0001***	0.08	[0.02 - 0.32]	***0.0003***	***< 0.0001***
Systemic inflammation	380	36.8%	2.94	[1.34 - 6.46]	***0.0074***	1.35	[0.63 - 2.93]	*0.4397*	***0.0232***
Parasite infection	380	27.4%	2.86	[0.98 - 8.35]	*0.0553*	0.85	[0.29 - 2.48]	*0.7649*	***0.0475***

Analyses are based on a logistic mixed model explaining the outcome with the intervention group, time of measurement, their interaction, age group, and sex as fixed effects; and with child ID and schools as random terms. The odds ratios (ORs) measure how the effect of the intervention group on the outcome is modified depending on the time of measurement, taking into account the interaction. ORs are presented with their 95% confidence intervals (CI). P-values are based on bilateral chi-square tests for the interaction.

Supplementary table 4: Baseline alpha-diversity metrics of the 380 children grouped according to sex, age, anthropometry, micronutrient and inflammatory status and parasitic infection.

	Variable		Kruskal-Wallis *H*	*q*-value
Shannon index	Sex	boy (n = 200) girl (n = 180)	2.132	0.144
	Underweight	yes (n = 100) no (n = 115)	2.864	0.091
	Stunting	yes (n = 172) no (n = 208)	0.233	0.629
	Anaemia	yes (n = 76) no (n = 304)	3.542	0.060
	Iron deficiency	yes (n = 193) vs. no (n = 187)	0.849	0.357
	Iron deficiency anaemia	yes (n = 47) no (n = 333)	2.771	0.096
	Hemoglobinopathy	yes (n = 211) no (n = 169)	2.226	0.136
	Zinc deficiency	yes (n=291) no (n=35)	0.009	0.925
	Systemic Inflammation	yes (n = 140) no (n = 240)	0.403	0.525
	Parasite infection	yes (n =104) no (n = 276)	2.126	0.145
	Gastrointestinal inflammation	yes (n = 10) no (n = 337)	0.393	0.531
Pielou's evenness	Sex	boy (n = 200) girl (n = 180)	0.966	0.326
	Underweight	yes (n = 100) no (n = 115)	3.474	0.062
	Stunting	yes (n=172) no (n=208)	0.021	0.884
	Iron deficiency	yes (n = 193) no (n = 187)	1.177	0.278
	Hemoglobinopathy	yes (n = 211) no (n = 169)	2.345	0.126
	Zinc deficiency	yes (n = 291) no (n = 35)	0.279	0.597
	Systemic Inflammation	yes (n = 140) no (n = 240)	0.276	0.600
	Parasite infection	yes (n = 104) no (n = 276)	1.517	0.218
	Gastrointestinal inflammation	yes (n = 10) no (n = 337)	0.016	0.898
Faith PD	Sex	boy (n = 200) girl (n = 180)	1.722	0.189
	Age	6-9 years (n =211) 10-14 years (n = 169)	0.190	0.663
	Underweight	yes (n = 100) no (n = 115)	0.449	0.503
	Stunting	yes (n = 172) no (n = 208)	0.089	0.765
	Anaemia	yes (n = 76) no (n = 304)	0.048	0.826
	Iron deficiency	yes (n = 193) no (n = 187)	0.086	0.770
	Iron deficiency anaemia	yes (n = 47) no (n = 333)	0.646	0.422
	Hemoglobinopathy	yes (n = 211) no (n = 169)	0.608	0.436
	Vitamin A deficiency	yes (n = 30) no (n = 350)	1.499	0.221
	Zinc deficiency	yes (n = 291) no (n = 35)	0.652	0.419
	Systemic Inflammation	yes (n = 140) no (n = 240)	0.951	0.330
	Parasite infection	yes (n = 104) no (n = 276)	0.074	0.785
	Gastrointestinal inflammation	yes (n = 10) no (n = 337)	1.883	0.170

Differences in alpha-diversity were estimated using a two-way Kruskal Wallis test. The number of samples in each subcategory is indicted in brackets. Only the variables with non-statistically significant differences are listed in the table (unadjusted P > 0.05).

Supplementary table 5: Baseline beta-diversity (pairwise distance) metrics of the 380 children grouped according to sex, age, anthropometry, micronutrient and inflammatory status and parasitic infection

	Variable		***Pseudo-F***	***q*-value**
Bray Curtis	Sex	boy (n = 200) girl (n = 180)	1.328	0.066
	Underweight	yes (n = 100) no (n = 115)	0.816	0.795
	Stunting	yes (n = 172) no (n = 208)	1.018	0.399
	Anaemia	yes (n = 76) no (n = 304)	1.196	0.159
	Iron deficiency anaemia	yes (n = 47) no (n = 333)	1.201	0.165
	Hemoglobinopathy	yes (n = 211) no (n = 169)	0.798	0.833
	Zinc deficiency	yes (n = 291) no (n = 35)	1.031	0.355
	Systemic Inflammation	yes (n = 140) no (n = 240)	1.234	0.130
	Parasite infection	yes (n = 104), no (n = 276)	0.849	0.748
	Gastrointestinal inflammation	yes (n = 10) no (n = 337)	1.024	0.410
Weighted UniFrac	Sex	boy (n = 200) girl (n = 180)	1.385	0.212
	Age	6-9 years (n =211) 10-14 years (n = 169)	1.654	0.126
	Underweight	yes (n = 100) no (n = 115)	0.730	0.603
	Stunting	yes (n = 172) no (n = 208)	0.658	0.648
	Anaemia	yes (n = 76) no (n = 304)	0.732	0.570
	Iron deficiency	yes (n = 193) no (n = 187)	1.281	0.268
	Iron deficiency anaemia	yes (n = 47) no (n = 333)	0.732	0.584
	Hemoglobinopathy	yes (n = 211) no (n = 169)	0.357	0.931
	Zinc deficiency	yes (n = 291) no (n = 35)	0.523	0.760
	Systemic Inflammation	yes (n = 140) no (n = 240)	1.017	0.380
	Parasite infection	yes (n = 104), no (n = 276)	0.533	0.767
	Gastrointestinal inflammation	yes (n = 10) no (n=337)	0.725	0.581
Unweighted UniFrac	Sex	boy (n = 200) girl (n = 180)	1.168	0.190
	Underweight	yes (n = 100) no (n = 115)	1.038	0.378
	Stunting	yes (n = 172) no (n = 208)	1.151	0.210
	Anaemia	yes (n = 76) no (n = 304)	0.849	0.736
	Iron deficiency	yes (n = 193) no (n = 187)	1.338	0.068
	Iron deficiency anaemia	yes (n = 47) no (n = 333)	0.985	0.452
	Hemoglobinopathy	yes (n = 211) no (n = 169)	0.798	0.827
	Vitamin A deficiency	yes (n = 30) no (n = 350)	1.111	0.281
	Zinc deficiency	yes (n = 291) no (n = 35)	1.326	0.092
	Systemic Inflammation	yes (n = 140) no (n = 240)	1.246	0.140
	Parasite infection	yes (n = 104), no (n = 276)	0.871	0.728
	Gastrointestinal inflammation	yes (n = 10) no (n = 337)	1.253	0.119
Jaccard	Underweight	yes (n = 100) no (n = 115)	0.963	0.625
	Stunting	yes (n = 172) no (n = 208)	1.114	0.096
	Anaemia	yes (n = 76) no (n = 304)	1.092	0.138
	Iron deficiency anaemia	yes (n = 47) no (n = 333)	1.142	0.060
	Hemoglobinopathy	yes (n = 211) no (n = 169)	0.988	0.532
	Vitamin A deficiency	yes (n = 30) no (n = 350)	1.121	0.095
	Zinc deficiency	yes (n = 291) no (n = 35)	1.056	0.208
	Systemic Inflammation	yes (n = 140) no (n = 240)	1.074	0.180
	Parasite infection	yes (n = 104), no (n = 276)	0.970	0.593
	Gastrointestinal inflammation	yes (n = 10) no (n = 337)	1.105	0.103

The number of samples in each subcategory is indicted in brackets. Differences in beta-diversity were estimated using a two-way PERMANOVA analysis with 999 permutations. Only variables with non-statistically significant differences are listed in the table.

Supplementary Table 6: Results of the linear mixed model analysis of LefSE-significant taxa,

Anaemia	ASV	Estimate	P value	Adjusted P value
Faecalibacterium	ASV4	0.55	0.018	0.0373
Limosilactobacillus	ASV13	0.85	0.013	0.0373
[Ruminococcus] torques group	ASV82	0.49	0.020	0.0373
Klebsiella	ASV88	0.63	0.014	0.0373
Erysipelatoclostridiaceae	ASV23	0.43	0.056	0.0877
Lachnospiraceae	ASV24	-0.01	0.938	0.9378
[Eubacterium] hallii group	ASV109	0.23	0.107	0.1474
Lachnospira	ASV300	-0.09	0.432	0.4748
Prevotellaceae NK3B31 group	ASV467	-0.1	0.270	0.3302
Prevotella_7	ASV390	0.57	0.006	0.0373
Anaerostipes	ASV210	0.45	0.015	0.0373

Iron deficiency anaemia		Estimate	P value	Adjusted P value
Faecalibacterium	ASV4	-0.76	0.007	0.030
Ruminococcus	ASV55	-0.66	0.031	0.058
HT002	ASV69	-0.59	0.033	0.058
[Eubacterium] hallii group	ASV109	-0.71	0.009	0.030
Lachnospira	ASV300	-0.44	0.010	0.030
Prevotella_7	ASV390	0.02	0.874	0.874
Prevotellaceae NK3B31 group	ASV467	0.02	0.863	0.874
Monoglobus	ASV475	-0.12	0.472	0.625
Lachnospiraceae UCG-010	ASV704	-0.05	0.682	0.779
Lachnospiraceae UCG-001	ASV1485	-0.11	0.250	0.400
Ruminococcaceae	ASV4.1	-0.70	0.001	0.021
Lachnospiraceae	ASV24	-0.57	0.011	0.030
Monoglobaceae	ASV475.1	-0.12	0.482	0.625
Oscillospirales	ASV4.2	-0.58	0.006	0.030
Lachnospirales	ASV24.1	-0.51	0.022	0.050
Monoglobales	ASV475.2	-0.11	0.508	0.625

Vitamin A deficiency		Estimate	P value	Adjusted P value
Romboutsia	ASV11	0.92	0.018	0.081
Roseburia	ASV89	-0.92	0.006	0.050
[Eubacterium] ruminantium group	ASV302	-0.23	0.246	0.277
UBA1819	ASV342	-0.26	0.148	0.226
[Eubacterium] siraeum group	ASV607	-0.27	0.111	0.226
Gordonibacter	ASV1300	0.10	0.347	0.347
Lactobacillaceae	ASV1	0.61	0.169	0.226
Lachnospiraceae	ASV24	-0.39	0.149	0.226
Desulfovibrionaceae	ASV1544	-0.18	0.176	0.226

Supplementary Table 6 (continued)

Systemic inflammation		Estimate	P value	Adjusted P value
Romboutsia	ASV11	-0.533	0.015	0.056
Enterococcus	ASV12	0.573	0.036	0.072
Succinivibrio	ASV30	-0.194	0.255	0.273
Alloprevotella	ASV43	0.269	0.187	0.216
Enterobacter	ASV77	-0.426	0.023	0.069
Lentilactobacillus	ASV79	-0.378	0.071	0.089
Klebsiella	ASV88	-0.384	0.071	0.089
CAG-352	ASV140	-0.497	0.009	0.056
Butyricimonas	ASV1245	0.125	0.048	0.072
Holdemania	ASV2404	0.113	0.000	0.005
Peptostreptococcaceae	ASV11.1	-0.412	0.043	0.072
Enterococcaceae	ASV12.1	0.578	0.032	0.072
Succinivibrionaceae	ASV30.1	-0.181	0.282	0.282
RF39	ASV70	-0.433	0.042	0.072
Gastranaerophilales	ASV97	-0.472	0.015	0.056

Parasitic infection		Estimate	P value	Adjusted P value
Holdemanella	ASV28	0.602	0.008	0.026
Weissella	ASV42	0.723	0.007	0.026
Sarcina	ASV93	0.489	0.004	0.026
Kurthia	ASV197	0.092	0.479	0.598
Fastidiosipila	ASV638	0.097	0.370	0.564
Gordonibacter	ASV1300	-0.089	0.174	0.349
UCG-004	ASV1899	0.000	0.991	0.991
Erysipelotrichaceae	ASV28.1	0.508	0.011	0.027
Hungateiclostridiaceae	ASV638.1	0.089	0.395	0.564
Desulfovibrionaceae	ASV1544	-0.047	0.561	0.624

The reference levels used in the table for each estimate are as follows: anaemia (no anaemia vs anaemia, with 'anaemia' as the reference level); iron deficiency anaemia (yes vs no, with 'yes' as the reference level); vitamin A deficiency (yes vs no, with 'yes' as the reference level); systemic inflammation (yes vs no, with 'yes' as the reference level); and parasitic infection (yes vs no, with 'yes' as the reference level). Significance was set at an adjusted (Benjamini-Hochberg) P-value of < 0.1.

Supplementary Table 7: Linear mixed model of alpha diversity measures.

Pielou's evenness	Estimate	Standard error	P value
Anaemia	0.047	0.018	0.009
Iron deficiency anaemia	-0.057	0.022	0.009
**Shannon diversity**			
Anaemia	0.247	0.106	0.021
Iron deficiency anaemia	-0.307	0.129	0.018

During the application of a linear mixed model, the reference level used for anaemia was established as "no anaemia", while for iron deficiency anaemia, the reference level was defined as the presence of iron deficiency anaemia. Only significant features (unadjusted $P < 0.05$) are shown
