# Supplementary material for: Faecal microbiota of schoolchildren is associated with nutritional status and markers of inflammation: a double-blinded cluster-randomized controlled trial using multi-micronutrient fortified rice
Source: Nat Commun. 2024 Jun 18;15:5204. doi: 10.1038/s41467-024-49093-4 (PMC11189458; doi:10.1038/s41467-024-49093-4)
Supplement: Supplementary file 3 — Reporting Summary [file 41467_2024_49093_MOESM3_ESM.pdf]

nature portfolio

Corresponding author(s): Christèle Humblot

Last updated by author(s): 2024/05/06

Reporting Summary

Nature Portfolio wishes to improve the reproducibility of the work that we publish. This form provides structure for consistency and transparency in reporting. For further information on Nature Portfolio policies, see our Editorial Policies and the Editorial Policy Checklist.

Statistics

For all statistical analyses, confirm that the following items are present in the figure legend, table legend, main text, or Methods section.

n/a	Confirmed	
☐	☒	The exact sample size (*n*) for each experimental group/condition, given as a discrete number and unit of measurement
☐	☒	A statement on whether measurements were taken from distinct samples or whether the same sample was measured repeatedly
☐	☒	The statistical test(s) used AND whether they are one- or two-sided<br>*Only common tests should be described solely by name; describe more complex techniques in the Methods section.*
☐	☒	A description of all covariates tested
☐	☒	A description of any assumptions or corrections, such as tests of normality and adjustment for multiple comparisons
☐	☒	A full description of the statistical parameters including central tendency (e.g. means) or other basic estimates (e.g. regression coefficient) AND variation (e.g. standard deviation) or associated estimates of uncertainty (e.g. confidence intervals)
☐	☒	For null hypothesis testing, the test statistic (e.g. *F*, *t*, *r*) with confidence intervals, effect sizes, degrees of freedom and *P* value noted<br>*Give P values as exact values whenever suitable.*
☒	☐	For Bayesian analysis, information on the choice of priors and Markov chain Monte Carlo settings
☒	☐	For hierarchical and complex designs, identification of the appropriate level for tests and full reporting of outcomes
☐	☒	Estimates of effect sizes (e.g. Cohen's *d*, Pearson's *r*), indicating how they were calculated

Our web collection on statistics for biologists contains articles on many of the points above.

Software and code

Policy information about availability of computer code

Data collection	Data entry, including quality checks and validation by double entry of questionnaires, was performed with EpiData version 3·1 (EpiData Association, Odense, Denmark). The bacterial 16S rRNA sequences were obtained the MiSeq platform (Illumina).
Data analysis	The DADA2 R package (version 1.20.0) was utilized to derive amplicon sequence variants (ASVs) from raw sequence data, following quality filtering and chimera removal. Taxonomy was also assigned using the DADA2 (version 1.20.0). Sequence alignment was conducted using the msa R package (version 1.34.0), and a phylogenetic tree was constructed with the phangorn R package (version 2.11.1). Data management and processing were performed using the phyloseq package (version 1.46.0). Alpha and beta diversity analyses were conducted using QIIME2 (version 2021.11) and the vegan R package (version 2.6.4). Statistical analyses and visualization were carried out in RStudio using R (version 4.1.2) with packages including ggplot2 (version 3.5.1), MaAsLin (version 1.12.0), microbiome (version 1.20.0), stats (version 4.3.2), and Tjazi (version 0.1.0.0). The functional potential of the microbial communities was predicted using PICRUSt2 (version 2.4.1). Group significance was evaluated using the Statistical Analysis of Metagenomic Profiles (STAMP, version 2.1.3), and differential abundance analyses were conducted with LEfSe on the Huttenhower Galaxy server (version 2.0, http://199.94.60.28/galaxy/). Logistic mixed models were employed to assess the impact of interventions on child growth and micronutrient status using R (version 4.1.0).<br>Logistic mixed models were performed to assess the impact of the intervention on child growth and micronutrient status using R 4.1.0.

For manuscripts utilizing custom algorithms or software that are central to the research but not yet described in published literature, software must be made available to editors and reviewers. We strongly encourage code deposition in a community repository (e.g. GitHub). See the Nature Portfolio guidelines for submitting code & software for further information.

Data

Policy information about availability of data

All manuscripts must include a data availability statement. This statement should provide the following information, where applicable:

- Accession codes, unique identifiers, or web links for publicly available datasets
- A description of any restrictions on data availability
- For clinical datasets or third party data, please ensure that the statement adheres to our policy

The 16SrRNA genes sequencing data generated in this study have been deposited in the National Centre for Biotechnology Information database under the accession number PRJNA882252 [https://www.ncbi.nlm.nih.gov/bioproject/882252]. The raw healthy volunteer-related clinical trial raw data, which could compromise protection of privacy of research participants are protected and are not available due to privacy laws. The processed data are provided with this paper in the Source Data file.
All codes have been deposited on GitHub repository https://github.com/yoh-s/FORISCA.

Research involving human participants, their data, or biological material

Policy information about studies with human participants or human data. See also policy information about sex, gender (identity/presentation), and sexual orientation and race, ethnicity and racism.

Reporting on sex and gender	Biological sex was determined in the study design based on self-report. Sex was considered on the study design since it has been previously shown that it can affects the faecal bacterial composition. 200 boys and 180 girls were included in this study and written informed consent was obtained from their parents or caretakers and verbal assent from the participating children prior to enrolment in the study, for sharing individual-level data. Link of sex with faecal bacterial alpha diversity, beta-diversity was investigated, and faecal bacterial biomarkers was researched.
Reporting on race, ethnicity, or other socially relevant groupings	Not applicable.
Population characteristics	Data on micronutrient status and faecal microbiota were available for 380 schoolchildren at baseline of the FORISCA study. The mean age of the children was 9.7 years (6-14 years old) and there were 53% of boys. Twenty percent of the children were anaemic, and 45% of the children were stunted. Prevalence of iron and zinc deficiencies were 51% and 89%, respectively, but the prevalence of vitamin A deficiency was lower (<8%). One-third of the children showed evidence of systemic inflammation and only 3% of the children had gastrointestinal inflammation. Parasites infection was detected in 27% of the children.
Recruitment	n total, six schools (out of 12 eligible) participating in the FORISCA project were selected for the present study. All schools were part of the WFP food program in Kampong Speu province. In each school, 11 boys and 11 girls from each class (level) were selected at random, from the list of students per class, hence, in total, there were 132 children per school (66 boys and 66 girls) selected for in-depth data collection.<br>There is a low level of selection bias in the current study, as school children were randomly selected from the list of names per level, before start of recruitment. However, bias might have occurred in the sense that all schools were WFP targeted schools, meaning that these schools serve the poorest segments of the population. Indeed, anemia prevalence in a control group of school children from schools not targeted by the WFP school meal program, was lower than in the placebo and intervention groups (Perignon et al Nutrients 2016, 8, 29; doi:10.3390/nu8010029).<br>This present study focusses on a subset of 380 children. A random selection was performed of children for whom all data were available at both points where stool samples had been collected for assessment of parasitic infection and gut inflammation status and who had not taken any antibiotics for the past three months.
Ethics oversight	This study was approved by the National Ethic Committee for Health Research (NECHR) of the Ministry of Health, Phnom Penh, Cambodia, the Ministry of Education, Youth and Sports, Phnom Penh, Cambodia, and the Research Ethics Committee of PATH, Seattle, USA. Written informed consent from participants has been obtained. The trial was registered at ClinicalTrials. gov (Identifier NCT0170641 [I https://classic.clinicaltrials.gov/ct2/show/record/NCT01706419?term=NCT01706419&draw=2&rank=1&view=record]).

Note that full information on the approval of the study protocol must also be provided in the manuscript.

Field-specific reporting

Please select the one below that is the best fit for your research. If you are not sure, read the appropriate sections before making your selection.

☒ Life sciences ☐ Behavioural & social sciences ☐ Ecological, evolutionary & environmental sciences

For a reference copy of the document with all sections, see nature.com/documents/nr-reporting-summary-flat.pdf

Life sciences study design

All studies must disclose on these points even when the disclosure is negative.

Sample size	This work focusses on a subset of 380 children from the FORISCA project, which was a large, double-blinded, cluster-randomized, placebo-

	controlled trial on the impact of fortified rice on the health and cognitive performance of 9,500 schoolchildren. A random selection was performed on children for whom all data were available at both collection points, and where stool samples had been collected for assessment of parasitic infection and gut inflammation status and who didn't take antibiotics for the past three months.
Data exclusions	Exclusion criteria included age below 6 and over 14 years, mental or physical disabilities, or severe anaemia (defined as haemoglobin concentration < 70 g/L). Children diagnosed with severe anaemia were provided with multiple micronutrient supplements for two months.
Replication	Considering the large number of participants, the study was not formally replicated.
Randomization	The six selected schools were randomly allocated to one of the three intervention groups using a computer-generated list with predefined criteria concerning the final size of the group.
Blinding	The trial was a full, double-blinded one. Researchers and subjects were blinded during the study as well as during the analyses of data.

Reporting for specific materials, systems and methods

We require information from authors about some types of materials, experimental systems and methods used in many studies. Here, indicate whether each material, system or method listed is relevant to your study. If you are not sure if a list item applies to your research, read the appropriate section before selecting a response.

Materials & experimental systems

n/a	Involved in the study	
☐	☒	Antibodies
☒	☐	Eukaryotic cell lines
☒	☐	Palaeontology and archaeology
☒	☐	Animals and other organisms
☐	☒	Clinical data
☒	☐	Dual use research of concern
☒	☐	Plants

Methods

n/a	Involved in the study	
☒	☐	ChIP-seq
☒	☐	Flow cytometry
☒	☐	MRI-based neuroimaging

Antibodies

Antibodies used	Antibodies used were from a kit for calprotectin measurement (Calpro AS, Norway). Sandwich enzyme-linked immunosorbent assay (ELISA) technique was used for measruement of Retinol-binding protein (RBP), ferritin (FER), soluble transferrin receptors (TfR), C-reactive protein (CRP), and α1-acid-glycoprotein (AGP) concentrations measurement (Erhardt et al 2004).
Validation	For Calprotectin measurement (Calpro AS, Norway), instructions from the manufacturer were used for validation. For Retinol-binding protein (RBP), ferritin (FER), soluble transferrin receptors (TfR), C-reactive protein (CRP), and α1-acid-glycoprotein (AGP) concentrations. RBP, FER, TfR, CRP, and AGP , the protocol published previsously with all validation was used (Erhardt, J.G.; Estes, J.E.; Pfeiffer, C.M.; Biesalski, H.K.; Craft, N.E. Combined measurement of ferritin, soluble transferrin receptor, retinol binding protein, and C-reactive protein by an inexpensive, sensitive, and simple sandwich enzyme-linked immunosorbent assay technique. J. Nutr. 2004, 134, 3127–3132, DOI: 10.1093/jn/134.11.3127).

Clinical data

Policy information about clinical studies

All manuscripts should comply with the ICMJEguidelines for publication of clinical research and a completedCONSORT checklist must be included with all submissions.

Clinical trial registration	The trial was registered at ClinicalTrials.gov (Identifier: NCT01706419).
Study protocol	The study protocol can be founf on ClinicalTrials.gov (Identifier: NCT01706419).
Data collection	The study was conducted in Kampong Speu, one of Cambodia's 23 provinces, situated 60 km west of Phnom Penh, the capital city, between November 2012 and July 2013.
Outcomes	Primary outcome measures were: anthropometry (weight, height and skinfold thickness) and micronutrient status (iron status (ferritin and soluble transferrin Receptor), zinc and vitamin A status (retinol-binding protein), using plasma concentrations), plasmatic biomarkers of inflammation (C-reactive protein (CRP) and α 1-acid-glycoprotein (AGP)).<br>The weight and height of the children were measured without footwear and wearing minimum clothing using standard procedures. Weight was measured once to the nearest 100g (model 881U scale; Seca, Hamburg, Germany). The accuracy of the scales was checked every day using a set of two calibration weights. Height was measured twice to the nearest 0.1 cm on a wooden stadiometer (UNICEF-Cambodia) and mean values were used. When differences between two measurements of height in the same child exceeded 0.5 cm, the measurements were repeated. Height-for-age Z-score (HAZ) and BMI-for-age Z-score (BAZ) were calculated according to the WHO 2006 reference. Underweight and stunting were defined as BAZ<−2 and HAZ<−2, respectively.<br>Non-fasting blood samples were taken from the antecubital vein in the morning following a standard protocol. Blood (5mL) was stored in trace-element free vacutainers with no anticoagulant (Vacuette, Greiner Bio One, Austria) at a temperature of < 5C. The blood samples were centrifuged (1,300g for 10 min) and serum samples were aliquoted and stored at -25°C until analysis. Iron status (ferritin and soluble transferrin receptor), vitamin A status (retinol-binding protein) and biomarkers of inflammation (C-reactive protein (CRP) and α 1-acid-glycoprotein (AGP)) were determined at VitMin laboratory (Willstaett, Germany). All these proteins were measured using a sandwich enzyme-linked immunosorbent assay (ELISA) technique. Zinc was measured at the National Institute of Nutrition (Hanoi, Vietnam) using a flame atomic absorption spectrophotometer (GBC, Avanta+) using trace element-free procedures. Faecal calprotectin was measured (Calpro AS, Norway) to estimate the gut inflammation.<br><br>Secondary outcome measures were: parasite infestation (number of parasite eggs in stools), gut inflammation (calprotectin concentrations in stool samples) and gut bacterial composition.<br>Faecal parasites were analysed as previously described using the Kato-Katz technique to determine helminth infection by the National Center for Parasitology, Entomology and Malaria control (CNM), Phnom Penh, Cambodia, and recorded as the number of eggs per gram of faeces. Faecal samples for taxonomic profiling of the gut microbiota were collected from the three groups at baseline and endline. Faecal samples were stored at -80°C until DNA extraction. DNA was extracted using the QIAamp DNA Stool Mini kit (ID: 51604, Qiagen, Les Ulis, France) according to the manufacturer's protocol. The V3-V5 hypervariable region of bacterial 16SrRNA was sequenced in paired-end mode (2×300 bp) on the MiSeq platform (Illumina, performed by the Research and Testing Laboratory in Lubbock, Texas, US) using primers 357F (5'-CCTACGGGAGGCAGCAG-3') and 926R (5'-CCGTCAATTCMTTTRAGT-3').

Plants

Seed stocks	Not applicable.
Novel plant genotypes	Not applicable.
Authentication	Not applicable.
